# PICO, PICOS and SPIDER: a comparison study of specificity and sensitivity in three search tools for qualitative systematic reviews

**DOI:** 10.1186/s12913-014-0579-0

**Published:** 2014-11-21

**Authors:** Abigail M Methley, Stephen Campbell, Carolyn Chew-Graham, Rosalind McNally, Sudeh Cheraghi-Sohi

**Affiliations:** University of Manchester, Centre for Primary Care, Williamson Building, Oxford Road, Manchester, M13 9PL UK; Institute of Primary Care and Health Sciences, Keele University, Keele, UK; Central Manchester Hospitals Site, Manchester Mental Health and Social Care Trust, Research and Innovation 3rd Floor, Rawnsley Building, Hathersage Road, Manchester, M13 9WL UK; NIHR Greater Manchester Primary Care Patient Safety Translational Research Centre, Institute of Population Health, The University of Manchester, Manchester, M13 9WL UK

**Keywords:** Health care, Users’ experiences, Multiple sclerosis (MS), Research evaluation, Research, Qualitative, Systematic reviews

## Abstract

**Background:**

Qualitative systematic reviews are increasing in popularity in evidence based health care. Difficulties have been reported in conducting literature searches of qualitative research using the PICO search tool. An alternative search tool, entitled SPIDER, was recently developed for more effective searching of qualitative research, but remained untested beyond its development team.

**Methods:**

In this article we tested the ‘SPIDER’ search tool in a systematic narrative review of qualitative literature investigating the health care experiences of people with Multiple Sclerosis. Identical search terms were combined into the PICO or SPIDER search tool and compared across Ovid MEDLINE, Ovid EMBASE and EBSCO CINAHL Plus databases. In addition, we added to this method by comparing initial SPIDER and PICO tools to a modified version of PICO with added qualitative search terms (PICOS).

**Results:**

Results showed a greater number of hits from the PICO searches, in comparison to the SPIDER searches, with greater sensitivity. SPIDER searches showed greatest specificity for every database. The modified PICO demonstrated equal or higher sensitivity than SPIDER searches, and equal or lower specificity than SPIDER searches. The modified PICO demonstrated lower sensitivity and greater specificity than PICO searches.

**Conclusions:**

The recommendations for practice are therefore to use the PICO tool for a fully comprehensive search but the PICOS tool where time and resources are limited. Based on these limited findings the SPIDER tool would not be recommended due to the risk of not identifying relevant papers, but has potential due to its greater specificity.

## Background

Systematic reviews are a crucial method, underpinning evidence based practice and informing health care decisions [1,2]. Traditionally systematic reviews are completed using an objective and primarily quantitative approach [3] whereby a comprehensive search is conducted, attempting to identify all relevant articles which are then integrated and assimilated through statistical analysis. The comprehensiveness of the search process has been viewed as a key factor in preventing bias and providing a true representation of available research [4]. Current research investigating the process of quantitative systematic reviews therefore focuses on methods for ensuring the most comprehensive and bias free searches possible [5]. Because of the time and resources required to complete a systematic and comprehensive search, efforts have been made to investigate the sensitivity of searches, and thus lessen the amount of time spent reviewing irrelevant articles with no benefit [6].

However, conducting comprehensive searches also forms the bedrock of qualitative or narrative reviews, now commonly referred to as qualitative evidence syntheses [7]. Qualitative evidence syntheses are now acknowledged as a necessary and valuable type of information to answer health services research questions [8]. However, difficulties in completing a sensitive yet comprehensive search of qualitative literature have been previously noted [9-11] including: poor indexing and use of key words of qualitative studies, the common use of titles that lack the keywords describing the article, and unstructured abstracts.

When devising a search strategy, a search tool is used as an organising framework to list terms by the main concepts in the search question, especially in teams where it is not possible to have an experienced information specialist as a member of the review team. The PICO tool focuses on the Population, Intervention, Comparison and Outcomes of a (usually quantitative) article. It is commonly used to identify components of clinical evidence for systematic reviews in evidence based medicine and is endorsed by the Cochrane Collaboration [2]. Due to its target literature base several of these search terms such as “control group” and “intervention” are not relevant to qualitative research which traditionally does not utilise control groups or interventions, and therefore may not appropriately locate qualitative research. However, these terms may become more relevant in the future as more trials and interventions incorporate qualitative research [12].

As the PICO tool does not currently accommodate terms relating to qualitative research or specific qualitative designs, it has often been modified in practice to “PICOS” where the “S” refers to the Study design [4], thus limiting the number of irrelevant articles.

Cooke et al. also addressed this issue of relevance by developing a new search tool entitled “SPIDER” (sample, phenomenon of interest, design, evaluation, research type), designed specifically to identify relevant qualitative and mixed-method studies [9]. The key features and differences of the SPIDER and PICO search tools are shown in Table 1. The addition of the “design” and “research type” categories to the SPIDER tool was intended to further increase the ability of this tool to identify qualitative articles, whilst removing irrelevant PICO categories such as the “comparison” group [9].

**Table 1:** **Search categories and SPIDER and PICO headings**

	**PICO**	**PICOS**	**SPIDER**
Multiple Sclerosis and patient/service user	**P**opulation	**P**opulation	**S**ample
Health care services	**I**ntervention	**I**ntervention	**P**henomenon of **I**nterest
Named types of qualitative data collection and analysis	**C**omparison	**C**omparison	**D**esign
Experiences, perceptions	**O**utcome	**O**utcome	**E**valuation
Qualitative or qualitative method	not applicable	**S**tudy type	**R**esearch type

Cooke et al. recommended that the SPIDER tool was tested further in qualitative literature searches [9]. Although it has been used previously in a scoping review to investigate gaps in an evidence base on community participation in rural health care [13], SPIDER has not yet been tested and evaluated in a qualitative systematic narrative review context. The authors of this article recently completed a systematic review of the qualitative research investigating experiences of health care services for people with Multiple Sclerosis [14]. On embarking on this review topic we faced many of the difficulties commonly discussed in identifying qualitative literature on a given topic, and identified SPIDER as a potential way of overcoming some of these difficulties. Therefore, the aim of this article was to test SPIDER by broadly replicating the work of Cooke et al. [9], specifically by comparing the two approaches: 1) the traditional PICO method of searching electronic databases with 2) the newly devised SPIDER tool, developed for qualitative and mixed-method research. In addition we wished to build and expand on the work of Cooke et al. [9] and so our third aim was to compare PICO and SPIDER to a modified PICO with qualitative study designs (PICOS, see Table 1 by investigating specificity and sensitivity across 3 major databases.

## Methods

### Inclusion and exclusion criteria

Studies eligible for inclusion were those that qualitatively investigated patients’ experiences, views, attitudes to and perceptions of health care services for Multiple Sclerosis. No date restriction was imposed on searches as this was an original review. Qualitative research, for this purpose, was defined by the Cochrane qualitative methods group [7] as using both a qualitative data collection method and qualitative analysis. Quantitative and mixed method studies were therefore excluded.

We define experience as “*Patients’ reports of how care was organised and delivered to meet their needs* p.301” [15]. Patients’ reports could refer to either experience of health care services delivery and organisation overall or their experiences of care by specific health care personnel. We included studies that investigated adults (aged 18 years old and older) with a diagnosis of Multiple Sclerosis, who had experience of utilising health care services at any time point. There were no restrictions on subtype of Multiple Sclerosis, gender, ethnicity or frequency of use of health care. Health care in this sense referred to routine clinical care (either state funded or privately funded) not trial protocols or interventions. Excluded studies included studies that focussed on self-management and studies that investigated quality of life.

Because of the focus on Multiple Sclerosis, studies were excluded if they used a mixed sample of various conditions (e.g. studies reported a mixed sample of people with neurological conditions) or if they used a sample of mixed respondents (i.e. people with Multiple Sclerosis and their carers) where results of patients with Multiple Sclerosis could not be clearly separated. If an article had a section or subtheme on health care services but this was not the main research area of the article, then that article was included; however only data from the relevant subtheme were extracted and included in the findings. Additional exclusion criteria were articles that only described carer or health care professional experiences not patient experiences. Conference abstracts, editorials and commentaries were not included.

### Search strategy

For this systematic search we developed a detailed search strategy in collaboration with a specialist librarian and information specialist. This search strategy was tailored to the three largest medical and nursing databases (Ovid MEDLINE, Ovid EMBASE, and EBSCO CINAHL Plus) as in Cooke et al.’s study [9] and search terms used a mixture of medical subject headings and keywords. To investigate the benefit of the SPIDER,PICO and PICOS tools we used identical search terms but combined them in different ways as shown in Tables 2, 3 and 4 below.

**Table 2:** **The search terms used in the SPIDER search**

**SPIDER tool** ^**a**^	**search terms**		
	**CINAHL plus**	**MEDLINE**	**EMBASE**
S	[MH Multiple Sclerosis OR TX multiple sclerosis] AND MH patients OR TX service user* or TX service-user*	[exp multiple sclerosis/OR multiple sclerosis.tw]AND [exp Patients/OR patient*.tw OR service user*.tw OR service-user*.tw OR exp consumer participation/OR consumer.tw]	[exp multiple sclerosis/OR multiple sclerosis.tw] AND [exp patient/patient$.tw OR service user$.tw OR service-user$ OR consumer$.tw]
P and I	MH (health services needs and demands) OR TX health care OR TX health services OR TX care OR MH patient care OR MH health personnel OR MH health services administration OR MH health services OR MH health facilities OR MH mental health services OR MH therapeutics OR TX specialist care MM “Multiple Sclerosis Psychosocial Factors” OR MM “Multiple sclerosis diagnosis” OR MM “Multiple sclerosis drug therapy”	exp “health care facilities, manpower and services”/OR health care.tw OR health services.tw OR exp Health Services Administration/OR exp Therapeutics/OR exp Diagnosis/OR organisations.tw OR exp Health Occupations/OR consultation.tw OR referral.tw OR exp Health Personnel/OR Health Education/OR hospital*.tw OR consultant*.tw OR neurologist*.tw OR doctor* OR practice nurse*.tw OR specialist nurse* OR psychologist*.tw OR general practitioner*.tw OR exp “psychiatry and psychology (non mesh)”/OR exp/Dentistry/ OR exp investigative techniques/OR exp “health care economics and organisations”/ OR specialist care.tw OR mental health services.tw OR mental health care.tw OR secondary care.tw	exp health care/OR health care.tw OR exp health service/OR exp health care organisation/OR exp health care utilization/ OR exp “care and caring”/OR care.tw OR medical care.tw OR exp health care personnel/OR health service$.tw OR health care professional$.tw OR exp health care quality/OR exp terminal care/OR exp health care management/ OR exp medical procedures/OR exp health care facility/OR hospital$.tw OR welfare/or *human needs/or *social welfare OR exp medical ethics/ OR consultant$.tw OR neurologist$.tw OR doctor$.tw OR practice nurse$.tw OR specialist nurse$.tw OR psychologist$.tw OR general practitioner$.tw OR mental health care.tw OR mental health services.tw or psycholog$ services.tw OR specialist care.tw OR secondary care.tw OR primary care.tw OR primary health care.tw
D	TX qualitative interview OR MH focus groups OR MH content analysis OR MH constant comparative method OR MH thematic analysis OR MH grounded theory OR MH ethnographic research OR MH phenomenological research OR MH semantic analysis OR TX interview*	exp interviews as Topic/OR exp Nursing Methodology Research/OR content analysis.tw OR constant comparative.tw OR grounded theory.tw OR ethography.tw OR interpretative phenomenological analysis.tw	exp interview/OR exp grounded theory/OR exp ethnography/OR interpretative phenomenological analysis.tw OR exp phenomenology/OR focus group$.tw OR exp content analysis/ OR exp thematic analysis/ OR exp constant comparative/
E	TX perception* OR MH patient satisfaction OR TX satisf* OR TX value* OR TX perceive* OR TX perspective* OR TX view* OR TX experience OR MH (health services needs and demand) OR TX opinion* OR MH consumer satisfaction OR TX belie* OR MM “Patient Attitudes” OR MM “Attitude to illness”	perceive*.tw OR perception*.tw OR exp Consumer Participation/OR *personal satisfaction/OR exp Consumer Satisfaction/OR satis*.tw OR exp Hospital-Patient Relations/OR exp Professional- Patient Relations/OR value*.tw OR perspective*.tw OR view*.tw OR experience*.tw OR need*.tw OR exp “Health Services Needs and Demand”/OR issue*.tw OR exp Attitude/OR belie*.tw OR opinion*.tw OR feel*.tw OR know*.tw OR understand*.tw	Perception$.tw OR exp satisfaction/OR satis$.tw OR value$.tw OR perceive$.tw OR exp psychological aspect/OR perspective$.tw OR view$.tw OR exp personal experience/OR experience$.tw OR exp health care need/OR need$.tw OR exp human needs/OR issue$.tw OR exp medical ethics/OR opinion$.tw OR exp attitude/OR exp health belief/OR attitude$.tw OR belie$.tw OR feel$.tw OR know$.tw OR understand$.tw
R	AB qualitative OR MH qualitative studies	exp Qualitative Research/ OR qualitative.tw	qualitative.tw OR qualitative analysis.tw OR exp qualitative research/

^a^[S AND P of I] AND [(D or E) AND R].

Footnote: * is a truncation symbol to retrieve terms with a common root within CINHAL Plus and MEDLINE. $ is a truncation symbol to retrieve terms with a common root within EMBASE.

**Table 3:** **The search terms used in the PICO search**

**PICO tool** ^**a**^	**Search terms**		
	**CINAHL plus**	**MEDLINE**	**EMBASE**
P	[MH Multiple Sclerosis OR TX multiple sclerosis] AND MH patients OR TX service user* or TX service-user*	[exp multiple sclerosis/OR multiple sclerosis.tw]AND [exp Patients/OR patient*.tw OR service user*.tw OR service-user*.tw OR exp consumer participation/ OR consumer.tw]	[exp multiple sclerosis/OR multiple sclerosis.tw] AND [exp patient/patient$.tw OR service user$.tw OR service-user$ OR consumer$.tw]
I	MH (health services needs and demands) OR TX health care OR TX health services OR TX care OR MH patient care OR MH health personnel OR MH health services administration OR MH health services OR MH health facilities OR MH mental health services OR MH therapeutics OR TX specialist care MM “Multiple Sclerosis Psychosocial Factors”OR MM “Multiple sclerosis diagnosis” OR MM “Multiple sclerosis drug therapy”	exp “health care facilities, manpower and services”/OR health care.tw OR health services.tw OR exp Health Services Administration/OR exp Therapeutics/OR exp Diagnosis/OR organisations.tw OR exp Health Occupations/OR consultation.tw OR referral.tw OR exp Health Personnel/OR Health Education/OR hospital*.tw OR consultant*.tw OR neurologist*.tw OR doctor* OR practice nurse*.tw OR specialist nurse* OR psychologist*.tw OR general practitioner*.tw OR exp “psychiatry and psychology (non mesh)”/OR exp/Dentistry/OR exp investigative techniques/OR exp “health care economics and organisations”/ OR specialist care.tw OR mental health services.tw OR mental health care.tw OR secondary care.tw	exp health care/OR health care.tw OR exp health service/OR exp health care organisation/OR exp health care utilization/ OR exp “care and caring”/OR care.tw OR medical care.tw OR exp health care personnel/OR health service$.tw OR health care professional$.tw OR exp health care quality/OR exp terminal care/ OR exp health care management/ OR exp medical procedures/OR exp health care facility/OR hospital$.tw OR welfare/or *human needs/or *social welfare OR exp medical ethics/OR consultant$.tw OR neurologist$.tw OR doctor$.tw OR practice nurse$.tw OR specialist nurse$.tw OR psychologist$.tw OR general practitioner$.tw OR mental health care.tw OR mental health services.tw or psycholog$ services.tw OR specialist care.tw OR secondary care.tw OR primary care.tw OR primary health care.tw
C	n/a	n/a	n/a
O	TX perception* OR MH patient satisfaction OR TX satisf* OR TX value* OR TX perceive* OR TX perspective* OR TX view* OR TX experience OR MH (health services needs and demand) OR TX opinion* OR MH consumer satisfaction OR TX belie* OR MM “Patient Attitudes” OR MM “Attitude to illness”	perceive*.tw OR perception*.tw OR exp Consumer Participation/ OR *personal satisfaction/OR exp Consumer Satisfaction/ OR satis*.tw OR exp Hospital-Patient Relations/OR exp Professional- Patient Relations/ OR value*.tw OR perspective*.tw OR view*.tw OR experience*.tw OR need*.tw OR exp “Health Services Needs and Demand”/OR issue*.tw OR exp Attitude/OR belie*.tw OR opinion*.tw OR feel*.tw OR know*.tw OR understand*.tw	Perception$.tw OR exp satisfaction/OR satis$.tw OR value$.tw OR perceive$.tw OR exp psychological aspect/OR perspective$.tw OR view$.tw OR exp personal experience/OR experience$.tw OR exp health care need/OR need$.tw OR exp human needs/OR issue$.tw OR exp medical ethics/OR opinion$.tw OR exp attitude/OR exp health belief/OR attitude$.tw OR belie$.tw OR feel$.tw OR know$.tw OR understand$.tw

^a^(P and I and O).

Footnote: * is a truncation symbol to retrieve terms with a common root within CINHAL Plus and MEDLINE. $ is a truncation symbol to retrieve terms with a common root within EMBASE.

**Table 4:** **The terms used in the PICOS search**

**PICO tool** ^**a**^	**Search terms**		
	**CINAHL plus**	**MEDLINE**	**EMBASE**
P	[MH Multiple Sclerosis OR TX multiple sclerosis] AND MH patients OR TX service user* or TX service-user*	[exp multiple sclerosis/OR multiple sclerosis.tw]AND [exp Patients/OR patient*.tw OR service user*.tw OR service-user*.tw OR exp consumer participation/ OR consumer.tw]	[exp multiple sclerosis/OR multiple sclerosis.tw] AND [exp patient/patient$.tw OR service user$.tw OR service-user$ OR consumer$.tw]
I	MH (health services needs and demands) OR TX health care OR TX health services OR TX care OR MH patient care OR MH health personnel OR MH health services administration OR MH health services OR MH health facilities OR MH mental health services OR MH therapeutics OR TX specialist care MM “Multiple Sclerosis Psychosocial Factors” OR MM “Multiple sclerosis diagnosis” OR MM “Multiple sclerosis drug therapy”	exp “health care facilities, manpower and services”/OR health care.tw OR health services.tw OR exp Health Services Administration/OR exp Therapeutics/OR exp Diagnosis/OR organisations.tw OR exp Health Occupations/OR consultation.tw OR referral.tw OR exp Health Personnel/OR Health Education/OR hospital*.tw OR consultant*.tw OR neurologist*.tw OR doctor* OR practice nurse*.tw OR specialist nurse* OR psychologist*.tw OR general practitioner*.tw OR exp “psychiatry and psychology (non mesh)”/OR exp/Dentistry/OR exp investigative techniques/ OR exp “health care economics and organisations”/OR specialist care.tw OR mental health services.tw OR mental health care.tw OR secondary care.tw	exp health care/OR health care.tw OR exp health service/OR exp health care organisation/ OR exp health care utilization/OR exp “care and caring”/OR care.tw OR medical care.tw OR exp health care personnel/OR health service$.tw OR health care professional$.tw OR exp health care quality/OR exp terminal care/OR exp health care management/OR exp medical procedures/OR exp health care facility/OR hospital$.tw OR welfare/or *human needs/or *social welfare OR exp medical ethics/OR consultant$.tw OR neurologist$.tw OR doctor$.tw OR practice nurse$.tw OR specialist nurse$.tw OR psychologist$.tw OR general practitioner$.tw OR mental health care.tw OR mental health services.tw or psycholog$ services.tw OR specialist care.tw OR secondary care.tw OR primary care.tw OR primary health care.tw
C	n/a	n/a	n/a
O	TX perception* OR MH patient satisfaction OR TX satisf* OR TX value* OR TX perceive* OR TX perspective* OR TX view* OR TX experience OR MH (health services needs and demand) OR TX opinion* OR MH consumer satisfaction OR TX belie* OR MM “Patient Attitudes” OR MM “Attitude to illness”	perceive*.tw OR perception*.tw OR exp Consumer Participation/OR *personal satisfaction/OR exp Consumer Satisfaction/ OR satis*.tw OR exp Hospital-Patient Relations/OR exp Professional- Patient Relations/OR value*.tw OR perspective*.tw OR view*.tw OR experience*.tw OR need*.tw OR exp “Health Services Needs and Demand”/OR issue*.tw OR exp Attitude/OR belie*.tw OR opinion*.tw OR feel*.tw OR know*.tw OR understand*.tw	Perception$.tw OR exp satisfaction/OR satis$.tw OR value$.tw OR perceive$.tw OR exp psychological aspect/OR perspective$.tw OR view$.tw OR exp personal experience/OR experience$.tw OR exp health care need/OR need$.tw OR exp human needs/OR issue$.tw OR exp medical ethics/ OR opinion$.tw OR exp attitude/OR exp health belief/OR attitude$.tw OR belie$.tw OR feel$.tw OR know$.tw OR understand$.tw
S	AB qualitative OR MH qualitative studies	Exp Qualitative Research/OR qualitative.mp AB qualitative OR MH qualitative studies	Qualitative.tw OR qualitative analysis.tw OR exp qualitative research/

^a^(P AND I AND C AND O AND S).

Footnote: * is a truncation symbol to retrieve terms with a common root within CINHAL Plus and MEDLINE. $ is a truncation symbol to retrieve terms with a common root within EMBASE.

One reviewer judged titles and abstracts against the inclusion criteria. If a title and abstract met the inclusion criteria then full text copies of all articles were retrieved for further investigation. Two authors reviewed these full text articles independently for relevance to the search aim (i.e. patients/service users with multiple sclerosis, experiences of health care services and qualitative research). Any disagreements were resolved via discussion. Data from included studies were extracted by both reviewers independently to ensure accuracy and then stored on a Microsoft Excel spread sheet. No ethical approval was required for this study.

## Results

All searches spanned from database inception until 12th October 2013. As in Cooke et al. [9], we reviewed our findings based on two metrics; the number of hits generated and of these, the number relevant to the search aim (see Table 5).

**Table 5:** **Hits generated and articles searched**

	**PICO search**		**PICO S search**		**SPIDER**	
	**Articles found in initial search**	**Articles included**	**Articles found in initial search**	**Articles included**	**Articles found in initial search**	**Articles included**
**Database**						
CINAHL plus	1350	After abstract and title =78	146	After abstract and title =56	146	After abstract and title =56
		After full review =14		After full review =12		After full review =12
EMBASE	14250	After abstract and title =35	189	After abstract and title =15	55	After abstract and title =9
		After full review =14		After full review =7		After full review =3
MEDLINE	8158	After abstract and title =34	113	After abstract and title =16	38	After abstract and title =14
		After full review =12		After full review =6		After full review =5

### Number of articles generated

As found in Cooke et al. [9], PICO created a much greater number of hits compared to SPIDER. A total of 23758 hits were generated using PICO, 448 hits were generated using PICOS and 239 hits were generated using SPIDER. Overall, the average reduction of hits (% across all three databases) was 98.58% for SPIDER vs. PICO, 97.94% for PICO vs. PICOS and 68.64% for PICOS vs. SPIDER. The time spent screening hits for relevant articles equated to weeks for the PICO hits and hours for the PICOS and SPIDER hits.

### Proportion of relevant articles

Articles which met the inclusion criteria after full text review are displayed in Table 6 [16-33]. Examination of the titles and abstracts of the identified articles resulted in the obtainment of 18 full text articles relevant at full text, across all databases and search tools.

**Table 6:** **Articles identified by database and search tool**

**Articles identified**		**Database (search tool used)**								
		**MEDLINE (PICO)**	**MEDLINE (PICOS)**	**MEDLINE (SPIDER)**	**EMBASE (PICO)**	**EMBASE (PICOS)**	**EMBASE (SPIDER)**	**CINAHL PLUS (PICO)**	**CINAHL PLUS (PICOS)**	**CINAHL PLUS (SPIDER)**
Lohne et al. [16].	The lonely battle for dignity	X	X	X	X	X		X	X	X
Mackereth et al. [17].	What do people talk about during reflexology	X			X					
Isaksson, and Ahlström [18].	Managing chronic sorrow	X	X	X	X	X		X	X	X
Edwards, Barlow & Turner [19].	Experiences of diagnosis and treatment among people with MS	X	X	X	X	X	X	X	X	X
Barker-Collo, Cartwright & Read [20].	Into the unknown: The experiences of individuals	X			X			X	X	X
Isaksson, & Ahlström [21].	From symptoms to diagnosis	X	X	X	X	X		X	X	X
Miller & Jezewski [22].	Relapsing MS patients experiences with galtiramer acetate	X	X	X	X	X	X	X	X	X
Johnson [23].	On receiving the diagnosis of ms	X			X					
Miller & Jezewski [24].	A phenomenologic assessment of relapsing MS patients’ experiences during treatment with Interferon Beta-1(*)	X	X		X	X		X	X	X
Miller [25].	The lived experience of relapsing ms	X			X			X	X	X
Aars & Bruusgaard [26].	Chronic disease and sexuality: An interview study	X			X					
Rintell et al. [27].	Patients’ perspectives on quality of mental health care	X			X			X		
Laidlaw & Henwood [28].	Patients with multiple sclerosis: Their experiences and perceptions of MRI				X	X	X			
Koopman & Schweitzer [29].	The journey to multiple sclerosis							X	X	X
Hansen, Krogh, Bangsgaard & Aabling [30].	Facing the diagnosis							X		
Loveland [31].	The experiences of African Americans and Euro-Americans with multiple sclerosis							X	X	X
Moriya & Suzuki [32].	A qualitative study relating to the experiences of people with MS							X	X	X
Classen & Lou [33].	Exploring rehabilitation and wellness needs of people with MS living in South Florida							X	X	X

#### PICO tool

For the PICO tool in CINAHL Plus, 5.78% of hits were deemed relevant after the title and abstract stage (78 articles/1350 articles), and 14/78 articles (17.95%) were confirmed to meet the inclusion criteria after full text review. For the PICO tool in MEDLINE, 0.42% of hits were deemed relevant after the title and abstract stage (34 articles/8158 articles) and 12/34 (35.29%) articles were confirmed to meet the inclusion criteria after full text review. For the PICO tool in EMBASE, 0.25% hits were deemed relevant after the title and abstract stage (35 articles/ 14250 articles) and 14/35(40%) articles were confirmed to meet the inclusion criteria after full text review.

#### PICOS tool

For the PICOS tool in CINAHL Plus, 38.36% of articles were relevant after the title and abstract stage (56 articles/146 articles) and 12/56 (21.43%) were confirmed to meet the inclusion criteria after full text review. For the PICOS tool in MEDLINE 14.16% of articles were relevant after the title and abstract stage (16 articles/ 113 articles) and 6/16 (37.5%) were confirmed to meet the inclusion criteria after full text review. For the PICOS tool in EMBASE 7.94% of articles were deemed relevant after the title and abstract stage (15 articles/189 articles) and 7/15 (46.67%) were confirmed to meet the inclusion criteria after full text review.

#### SPIDER tool

For the SPIDER tool in CINAHL Plus 38.36% of articles were relevant after the title and abstract stage (56 articles/146 articles) and 12/56 (21.43%) were confirmed to meet the inclusion criteria after full text review. For the SPIDER tool in MEDLINE, 36.81% hits were deemed relevant at the title stage (14 articles/38 articles) and 5/14 articles (35.71%) were confirmed to meet the inclusion criteria after full text review. For the SPIDER tool in EMBASE, 16.36% were relevant at the title stage (9 articles/55 articles) and 3/9 (33.33%) were confirmed to meet the inclusion criteria after full text review.

#### Sensitivity and specificity

The SPIDER tool identified 13 relevant articles out of 239 articles across all three databases (5.43%) compared to PICOS which identified 13 articles out of 448 articles (2.90%) and PICO which identified 18 articles out of 23758 articles (0.076%). Of the 18 relevant articles identified by the PICO tool, 66.66% came from both MEDLINE and CINAHL Plus (12 articles each), and 72.22% came from EMBASE (13 articles). Of the 13 relevant articles identified by the PICOS tool 46.15% came from MEDLINE (6 articles), 53.84% came from EMBASE (7 articles) and 92.31% came from CINAHL Plus (12 articles). Of the 13 relevant articles identified by SPIDER, 38.46% came from MEDLINE (5 articles) and 23.07% came from EMBASE (3 articles) and 92.30% came from CINAHL Plus (12 articles) Table 7.

**Table 7:** **Sensitivity and specificity for each search tool by database**

**Search tool and database**	**Sensitivity (% relevant texts identified out of all relevant hits)**	**Specificity (% relevant texts identified out of all hits)**
CINAHL PICO	14/18 = 77.78	14/1350 = 1.04
CINAHL PICO S	12/18 = 66.67	12/146 = 8.22
CINAHL SPIDER	12/18 = 66.67	12/146 = 8.22
MEDLINE PICO	12/18 66.67	12/8158 = 0.15
MEDLINE PICO S	6/18 = 33.33	6/113 = 5.32
MEDLINE SPIDER	5/18 = 27.78	5/14 = 35.71
EMBASE PICO	13/18 = 72.22	14/14250 = 0.1
EMBASE PICO S	7/18 = 38.88	7/189 = 3.7
EMBASE SPIDER	3/18 = 16.67	3/55 = 5.45

Different articles were found across different tools and databases (as shown in Table 6). All three databases were checked for all articles. One article was available in CINAHL Plus but not identified by any of the tools [17]. Two papers were identified in all databases through all search tools. Five papers were identified in MEDLINE through all search tools, three identified in EMBASE through all search tools and 12 identified in CINAHL through all search tools. Five papers were identified solely in CINAHL Plus, with one of these papers only identified using the PICO search method. One paper was identified by all search tools in EMBASE but not identified by any in MEDLINE. No new studies were identified using the SPIDER or PICOS tools alone in any database.

## Discussion

In this article we addressed the aim of replicating a comparison between the SPIDER, PICOS and PICO search tools. As previously described in Cooke et al. [9], the SPIDER tool produced a greatly reduced number of initial hits to sift through, however in this study it missed five studies that were identified through the PICO method. This may be partly be explained by the nature of the research question prompting the search. As this study included subthemes of studies whose focus differed from the initial research question (i.e. only a smaller section of the paper related to health care) then it’s possible that these studies were picked up by a broader search but not the highly specific SPIDER search. Other authors researching the process of qualitative literature reviews have previously commented that there appears to be a decision to be made about the benefits of comprehensiveness of findings versus the accuracy of the studies identified [11]. Given the common nature of using sub-sections of papers for systematic reviews then our findings suggest that comprehensiveness needs to be the key for this type of search.

The PICOS tool was more specific than the PICO tool, but did not identify any additional relevant hits to the SPIDER tool, suggesting it is of approximately equal sensitivity. PICOS identified the same number of papers as the SPIDER tool and both demonstrated a substantially lower number of hits generated than a regular PICO search. The SPIDER tool showed the greatest specificity due the small number of hits generated. This may mean that review teams with very limited resources or time, and who are not aiming for a totally comprehensive search (i.e. in the case of scoping studies), would benefit from using the SPIDER tool. This might be applicable particularly to studies such as qualitative syntheses, where the research aim is theoretical saturation, not a comprehensive search [34]. In addition, articles written to influence policy often require swift publication, providing another area in which either SPIDER or PICOS might improve current practice.

The issue of time was also related to the number of relevant articles identified per database. Whilst EMBASE generated nearly twice as many hits as MEDLINE, only one additional paper was found. The PICO tool identified all articles, suggesting that where time is not a factor, it might be of more benefit to use this tool, as SPIDER demonstrated lower sensitivity, did not identify any new articles and identified fewer relevant articles than PICO.

Our findings indicate that it is worthwhile testing a chosen search tool across various databases as they produce different results; i.e. CINHAL Plus identified papers not identified in MEDLINE or EMBASE databases. It is therefore important for future research to investigate the potential of the SPIDER vs. PICOS and PICO tools as a base for the recommended comprehensive searching process, by investigating the contribution of the SPIDER and PICOS tools at every stage from the initial search hits, to the final included relevant articles.

As CINAHL is a database dedicated to nursing and allied health research, it was expected that it would produce a greater number of relevant articles than more medically focussed databases [10], as nursing and allied areas have traditionally been at the forefront of qualitative investigations into Multiple Sclerosis.

SPIDER proved to be a tool designed to formulate search terms easily, as it naturally fits the crucial elements of the search question. However, even though some qualitative keywords are necessary to identify qualitative studies, including the words “*qualitative research*” AND the name of the type of research e.g. “*grounded theory*” might be too restrictive, particularly given the poor use of the qualitative index term, and might partially explain the fewer studies identified by SPIDER in comparison to PICO. Studies not identified by the SPIDER model in MEDLINE and EMBASE databases did not use keywords such as “*qualitative*”, but some described qualitative methods, such as “*phenomenological-hermeneutic*” [16] or “*interview(s)*” [20,23].

In all PICO searches for MEDLINE and EMBASE the word “*qualitative*” combined with the phrase “*multiple sclerosis*” identified many quantitative studies reporting brain scan assessments that were wholly unrelated to the search aim. This was because the word “*qualitative*” in this context referred to using a qualitative method to provide information about the quality of the scan and any potential flaws [35]. This caused a problem with specificity, resulting in thousands of inappropriate hits as there was no way to exclude studies with the word “*qualitative*” unless all articles clearly utilised and indexed qualitative research methods in the title, abstract and keywords.

Many studies were excluded at the full text stage on the basis that the samples were mixed: being comprised of either various neurological conditions or mixed groups of people i.e. patients and carers/patients and health care professionals and so forth. Without clearer titles and abstracts, and potentially an indexing phrase that indicates mixed samples, there is no way of avoiding this issue. Excluding the phrases “*caregivers*” or “*health care professionals*” would have excluded any studies that used these phrases (for example in the introduction or implication for future research sections) and therefore it is difficult to see how this could be prevented. A strength and limitation of our study is that whilst it details a real world example of evidence searching, it only addresses one topic. Further research should test these search tools against a wider variety of narrative review and meta-synthesis topics.

## Conclusions

SPIDER greatly reduced the initial number of articles identified on a given search due to increased specificity, however because of lower sensitivity omitted many relevant papers. The PICOS tool resulted in an overall more sensitive search, but still demonstrated poor specificity on this topic. Further investigations of the specificity and sensitivity of SPIDER and PICOS on varied topics will be of benefit to research teams with limited time and resources or articles necessary to impact on policy or change current practice. However, where comprehensiveness is a key factor we suggest that the PICO tool should be used preferentially. Part of the lower identification rate for SPIDER (in comparison to PICO) was poor labelling and use of qualitative keywords in indexing studies. As both individual research submissions and journal/database indexers improve, or standardise, the indexing of qualitative studies, it is likely that the relevance of the SPIDER tool will increase. The recommendation for current practice therefore is to use the PICO tool across a variety of databases. In this article we have shown that SPIDER is relevant for those researchers completing systematic narrative reviews of qualitative literature but not as effective as PICO. Future research should investigate the use of SPIDER and PICOS across varied databases.
